# Difference analysis of multidimensional electroencephalogram characteristics between young and old patients with generalized anxiety disorder

**DOI:** 10.3389/fnhum.2022.1074587

**Published:** 2022-11-24

**Authors:** Jie Wang, Jiaqi Fang, Yanting Xu, Hongyang Zhong, Jing Li, Huayun Li, Gang Li

**Affiliations:** ^1^Key Laboratory of Urban Rail Transit Intelligent Operation and Maintenance Technology and Equipment of Zhejiang Province, Zhejiang Normal University, Jinhua, China; ^2^College of Mathematics and Computer Science, Zhejiang Normal University, Jinhua, China; ^3^College of Engineering, Zhejiang Normal University, Jinhua, China; ^4^College of Foreign Language, Zhejiang Normal University, Jinhua, China; ^5^College of Teacher Education, Zhejiang Normal University, Jinhua, China; ^6^Key Laboratory of Intelligent Education Technology and Application, Zhejiang Normal University, Jinhua, China; ^7^College of Mathematical Medicine, Zhejiang Normal University, Jinhua, China; ^8^Key Laboratory for Biomedical Engineering of Ministry of Education of China, Department of Biomedical Engineering, Zhejiang University, Hangzhou, China

**Keywords:** generalized anxiety disorder (GAD), age, electroencephalogram (EEG), power spectral density (PSD), fuzzy entropy (FE), functional connectivity (FC), machine learning

## Abstract

Growing evidences indicate that age plays an important role in the development of mental disorders, but few studies focus on the neuro mechanisms of generalized anxiety disorder (GAD) in different age groups. Therefore, this study attempts to reveal the neurodynamics of Young_GAD (patients with GAD under the age of 50) and Old_GAD (patients with GAD over 50 years old) through statistical analysis of multidimensional electroencephalogram (EEG) features and machine learning models. In this study, 10-min resting-state EEG data were collected from 45 Old_GAD and 33 Young_GAD. And multidimensional EEG features were extracted, including absolute power (AP), fuzzy entropy (FE), and phase-lag-index (PLI), on which comparison and analyses were performed later. The results showed that Old_GAD exhibited higher power spectral density (PSD) value and FE value in beta rhythm compared to theta, alpha1, and alpha2 rhythms, and functional connectivity (FC) also demonstrated significant reorganization of brain function in beta rhythm. In addition, the accuracy of machine learning classification between Old_GAD and Young_GAD was 99.67%, further proving the feasibility of classifying GAD patients by age. The above findings provide an objective basis in the field of EEG for the age-specific diagnosis and treatment of GAD.

## Introduction

Generalized anxiety disorder (GAD) is usually described as frequent/persistent, accompanied by persistent significant nervousness, characterized by autonomic nervous function excitement and excessive vigilance (Bebbington and Jacoby, 1986; Tyrer and Baldwin, 2006; Mah et al., 2016; Buff et al., 2018; Santomauro et al., 2021). According to the Lancet report on anxiety disorders in 2021, the number of anxiety patients has increased by 25.6% from the estimated 298 million in 2020 to the actual 374 million in 2021 (Santomauro et al., 2021). As a subtype of anxiety disorder (Buff et al., 2018; Xu et al., 2021), GAD had a prevalence rate in adults between 4 and 7% (Huang et al., 2018), seriously affecting the normal life of individuals and families. Moreover, studies have reported that Old_GAD patients were quite different from Young_GAD patients in clinical presentations (Skoog, 2011; Altunoz et al., 2018), severity (Flint, 2005, 2009), and treatment responses (Wetherell et al., 2003; Lenze et al., 2009; Thorp et al., 2009). When the same diagnostic and therapeutic criteria are used for Young and Old_GAD patients, there is a high probability of clinical misdiagnosis and mistreatment. Therefore, the classification of GAD patients by age has important clinical value. Although some progress has been made in the psychophysiology of GAD in recent years (Grillon and Buchsbaum, 1987; Oathes et al., 2008; Smith et al., 2016; Wang et al., 2016; Pang et al., 2019), there is little research on the electrophysiological mechanism of GAD in different age groups.

Recently, a great variety of neuroimaging technologies have been used to reveal the neural activity of mental disorders, including functional Magnetic Resonance Imaging (fMRI) (Wang et al., 2016), electromagnetic tomography (ETA) (Clancy et al., 2020), and electroencephalogram (EEG) (Newson and Thiagarajan, 2019; Fusina et al., 2022). Among these neurotechnical means, EEG is a non-invasive method to obtain electrophysiological signals, and has a high temporal resolution, which can directly measure the neural activity of the participant’s brain, describe the functional changes during its dynamic activities, and reflect the mental state of the participant. Therefore, EEG has been widely used in the study of neural mechanisms and clinical diagnosis of mental diseases [e.g., depression (Feng et al., 2012; Jesulola et al., 2015; Steiger and Pawlowski, 2019), anxiety disorder (Tyrer and Baldwin, 2006; Olatunji et al., 2010; Hilbert et al., 2014), sleep disorder (Papadimitriou et al., 1988; Huang et al., 2019), and epilepsy (Mporas et al., 2015)]. Common EEG analysis methods include power spectral density (PSD) analysis (Fenton et al., 1980; Pradhan and Dutt, 1994) [e.g., absolute power (AP), relative power, and power ratio], non-linear dynamics analysis (Nikias and Petropulu, 1993; Alotaibi et al., 2022) [e.g., approximate entropy, fuzzy entropy (FE), and sample entropy], and brain functional connectivity (FC) analysis (Stam et al., 2007; Piho and Tjahjadi, 2020; Li et al., 2022) [e.g., Partial directed coherence (PDC), mutual information (MI), and phase-lag-index (PLI)]. In this study, the PSD, FE, and PLI were used as the main methods to analyze EEG data and to reveal the electrophysiological differences of GAD in different age groups.

Although researchers are increasingly interested in the brain pathogenesis of mental diseases, few studies have focused on the brain electrophysiological mechanism of different ages with GAD. Research have shown that the structure and function of the brain change with aging (Choi et al., 2020; Seoane et al., 2022), at the same time, senescence causes a decline in cognitive level (Tian et al., 2018) and cortical coordination (Blackmon et al., 2011). Mohlman et al. (2017) verified that the slow-down EEG markers can be reliably extracted from prefrontal EEG in the elderly. Despite a lack of research on EEG in patients with GAD at different ages, relevant psychological studies have revealed that Old_GAD patients are significantly different from Young_GAD patients in terms of symptom expression (Miloyan et al., 2014), and the anxiety symptoms are related to the changes in the beta rhythm of PSD value (Grin-Yatsenko et al., 2009). Meanwhile, anxiety disorder also changes the brain structure and function (Sylvers et al., 2011; Imperatori et al., 2019), which was related to several neural abnormalities [e.g., EEG desynchronization, abnormal resting state (Knyazev et al., 2004; Aftanas and Pavlov, 2005), and emotional stimulation]. Previous research on the brain FC of anxiety disorder has demonstrated that the prefrontal limbic connectivity of Old_GAD patients is enhanced during anxiety (Mohlman et al., 2017), the connectivity patterns of GAD patients at different ages are different (Massullo et al., 2020), and the correlation between age and anxiety is highlighted in the frontal region of the brain (Mohlman et al., 2017). Based on former findings, this study attempts to reinforce the studies on Young_GAD and Old_GAD through multi-channel EEG.

In this study, multidimensional EEG features (including AP, FE, and PLI) were used to expand the early research on the neural mechanism of anxiety disorders through a single linear or non-linear feature. Furthermore, machine learning models were used to further verify GAD by age. The purpose of the present study is to enhance the understanding of the underlying differential neural mechanisms of Old_GAD and Young_GAD in multidimensional EEG features. Specifically, this study used multidimensional EEG features, combined with traditional one-way analysis of variance (ANOVA), to investigate the significant differences between Old_GAD and Young_GAD in the PSD, FE, and PLI features. It is intended to reveal the neurodynamic mechanism of Old_GAD and Young_GAD, so as to contribute to the clinical triage of GAD patients by age.

## Materials and methods

### Participants

Seventy-eight GAD patients were recruited from the local Hospital, and their age ranges from 22 to 68 years old. All patients met the diagnostic criteria for GAD in the fourth revision of The Diagnostic and Statistical Manual of Mental Disorders (DSM-IV) (Pull, 1995). The Hamilton Anxiety Scale (HAMA) was used to evaluate anxiety symptoms, and the scores of patients were ≥17. Meanwhile, the inclusion criteria which all subjects need to meet were as follows, (1) Right-handed. (2) No other mental disorders except GAD (e.g., dementia, schizophrenia, epilepsy, delusional disorder, bipolar disorder, and depression disorder) and physical disorders (e.g., severe cardiopulmonary, hepatorenal insufficiency, malignant tumor, and autoimmune diseases). (3) No history of substance and alcohol abuse. (4) No history of brain damage. (5) Enough sleep the day before data collection and no smoking, no coffee, or strong tea for 8 h.

Based on the literature research (Le Roux et al., 2005; Vogelzangs et al., 2013; Gayete et al., 2020), we divided GAD patients into Old_GAD and Young_GAD according to the standard of a 50-year-old. Table 1 shows the demographic and clinical characteristics of the two groups. Wherein the Young_GAD included 33 participants, with ages ranging from 22 to 48 years old (37.36 ± 7.46), including 8 males and 25 females, and the score of HAMA was 24.39 ± 8.81. And the Old_GAD included 45 participants, with ages ranging from 50 to 68 years old (56.09 ± 4.60), including 13 males and 22 females, and the score of HAMA was 25.16 ± 7.43. There was no significant difference in age and HAMA scores between the two groups.

**TABLE 1:** Demographic and clinical characteristics of the participants.

Characteristics	Young_GAD	Old_GAD	*P*-value
Number	33	45	–
Gender: male/female	8/25	13/32	–
Age (year)	22–48 (37.36 ± 7.46)	50–68 (56.09 ± 4.60)	7.4e × 10^–28^
HAMA	24.39 ± 8.81	25.16 ± 7.43	0.68

### Electroencephalogram data acquisition and preprocessing

Each subject was required to collect 10 min of resting-state EEG data and was asked to remain awake, eyes closed, and in a relaxed state during the collection. Data acquisition was performed in the professional EEG room of the hospital. The EEG model was Nicolet EEG TS215605, and its parameters were set as follows. (1) According to the international standard 10–20 electrode arrangement system, 16 electrodes were selected, including FP1, FP2, F3, F4, C3, C4, P3, P4, O1, O2, F7, F8, T3, T4, T5, and T6 (The grounding electrode position is Fpz). (2) Left and right mastoid as reference electrodes. (3) Sampling rate was set to 250 Hz. (4) The electrode impedance should be less than 5 *K*Ω.

The collected EEG data was preprocessed according to the following steps. (1) Down-sampled the EEG data to 125 Hz, and used the fourth-order Butterworth bandpass filter from 4 to 30 Hz. (2) Used fast independent component analysis (ICA) to remove EEG artifacts, such as eyes blinking, electrocardiogram (EKG), electromyography (EMG), and so on. (3) Took 4 s of continuous EEG data (50% overlap) for EEG segmentation to obtain 7,991 Young_GAD data samples and 9,725 Old_GAD data samples. (4) Extracted the EEG rhythms of θ (4–8 Hz), α1 (8–10 Hz), α2 (10–13 Hz), and β (13–30 Hz) of each EEG sample through the same bandpass filter.

### Multi-dimensional electroencephalogram characteristics extraction

The PSD, FE, and PLI have been demonstrated to be effective and feasible for detecting anxiety disorders (Shen et al., 2022). These three methods decode EEG information from three different dimensions, which portrayed the neurophysiological implications of EEG signals from three different perspectives. In this study, these features were applied to reveal the electrophysiological differences between Old_GAD and Young_GAD. For each sample of the EEG data, the calculated EEG features was shown in Table 2.

**TABLE 2:** The electroencephalogram (EEG) features were calculated in this study.

	AP	FE	PLI
Numbers	4 16	4 16	4 16 (16-1)/2

We used 16 EEG channels and four frequency bands. The absolute power (AP) and fuzzy entropy (FE) were calculated based on a single channel, and phase-lag-index (PLI) was computed between two EEG channels.

#### Absolute power extraction

In this study, the AP of each sample was calculated using the periodogram method. For the given EEG signal *X*(n), its frequency spectrum *X*_*N*_(f) can be estimated by Fast Fourier Transform (FFT). Then, the power spectrum *P*_*x*_(f) is obtained from the modulo square of the spectrum, as in Eq. 1. The EEG power of each rhythm can be derived from Eq. 2. *E*(*h*) is the power value of the *h* rhythm and the *f_h_* and *f_l_* are the upper and lower frequency limits of the *h* rhythm, respectively.

For the *N* observations of the EEG signals *x*(*n*) of each channel using FFT to obtain the spectrum *X*_*N*_(f), the power spectrum *P*_*x*_(f) can be defined as Eq. 1.


(1) $$P_{x}\,\left(f\right)=\frac{1}{N}\,{\left|X_{N}\,\left(f\right)\right|}^{2}$$

The power E(*h*) of each rhythm *h* of EEG is defined as Eq. 2.


(2) $$E\,\left(h\right)=\frac{1}{f_{h}-f_{l}}\,\int_{f_{l}}^{f_{h}}P_{x}\,\left(f\right)\,df$$

where *f_h_* and *f_l_* are the upper limit and lower limit of the frequency of *h* rhythm, respectively.

#### Fuzzy entropy calculation

Given an EEG signal *x*(i)(*i* = 1,2,,N) with length N and reconstructed the signal into m-dimensional space *R_m_* as Eq. 3.


(3) $$R_{i}^{m}=\left\{x\,\left(i\right),x\,\left(i+1\right),\ldots ,x\,\left(i+m-1\right)\right\}-x_{0}\,\left(i\right)$$

(i=1,,N-m+1)

where {*x*(*i*),*x*(*i* + 1),…,*x*(*i* + *m*1)} represents *m* consecutive sampling points starting from the *i*-th sampling point, and *x*_0_(*i*) represents the average of the m sampling point as Eq. 4.


(4) $$x_{0}\,\left(i\right)=\frac{1}{m}\,\sum_{j=0}^{m-1}x\,\left(i+j\right)$$

The maximum distance $d_{ij}^{m}$ between the m-dimensional reconstruction vectors $R_{i}^{m}$ and $R_{j}^{m}$ is defined as Eq. 5.


(5) $$d_{i\,j}^{m}=d\,\left[R_{i}^{m},R_{j}^{m}\right]$$

$$=m\,a\,x\,\left\{\left|x\,\left(i+k\right)-x_{0}\,\left(i\right)-x\,\left(j+k\right)-x_{0}\,\left(j\right)\right|\right\}$$

k∈(0,m-1),i≠j

Given *n* and *r*, the similarity degree $D_{ij}^{m}$ between $R_{i}^{m}$ and $R_{j}^{m}$ is calculated by using the fuzzy function $\mu \,\left(d_{i\,j}^{m},n,r\right)$ as Eq. 6.


(6) $$D_{i\,j}^{m}=\mu \,\left(d_{i\,j}^{m},n,r\right)=\exp\left(-\frac{{\left(d_{i\,j}^{m}\right)}^{n}}{r}\right)$$

where *n* and *r* are the gradient and width of the boundary of the exponential function, respectively. Thus, define function φ*^m^*(*n*,*r*) as the average of all similarity degree of all adjacent vectors $R_{j}^{m}$ for each vector $R_{i}^{m}$ (*j*≠*i*) as Eq. 7.


(7) $$\varphi^{m}\,\left(n,r\right)=\frac{1}{N-m}\,\sum_{i=1}^{N-m}\left(\frac{1}{N-m-1}\,\sum_{j=1,j\neq i}^{N-m}D_{i\,j}^{m}\right)$$

repeating the calculation process from Eqs 3–7, a vector φ^*m* + 1^(*n*,*r*) of *m* + 1 dimension is obtained. Finally, the FE is evaluated as Eq. 8.


(8) $$\text{FuzzyEn}\,\left(m,n,r\right)=\underset{N\to \infty }{\text{lim}}\left[\ln\varphi^{m}\,\left(n,r\right)-\ln\varphi^{m+1}\,\left(n,r\right)\right]$$

when the EEG signal is of finite length N, the FE can be defined as Eq. 9.


(9) $$\text{FuzzyEn}\,\left(m,n,r,N\right)=\left[\ln\varphi^{m}\,\left(n,r\right)-\ln\varphi^{m+1}\,\left(n,r\right)\right]$$

Four parameters need to be fixed when calculating FE. They are signal length N, embedding dimension m and the gradient n and width r of the exponential function boundary. In this study, the length N of our EEG signal *x*(*i*) was 1,000, and the selected embedding dimension m is 2, which is a typical value. In addition, when gradient n tends to infinity, the information near the boundary point is seriously lost (Chen et al., 2007). Therefore, n should be a small positive integer such as 2 and 3. The smaller *r* value is susceptible to noise, while the larger *r* value may lead to the loss of useful information. The r is set to *k* times of the standard deviation of the EEG signal *x*(*i*), and the common range of k is 0.1 ≤ *k* ≤ 0.25 (Shen et al., 2022). In this study, the *n* and *k* were set to 2 and 0.2, respectively.

#### Phase-lag-index calculation

The PLI is an index of the asymmetry in the distribution of relative phase calculated from an instantaneous phase of two-time series (the signals of a pair of EEG electrodes). Given the paired time series *x*_1_(*t*) and *x*_2_(*t*) that have passed the band-pass filtering, and the Hilbert transform used to calculate the instantaneous phase *z*_*i*_(*t*) (Moezzi et al., 2019) as Eq. 10.


(10) $$z_{i}\,\left(t\right)=x_{i}\,\left(t\right)+j\,\frac{1}{\pi }\,P.V.\int_{\text{∞}}^{\infty }\frac{x_{i}\,\left(t\right)}{t-\tau }\,d\tau$$

where, *P.V.* represents Cauchy principal value. The relative phase of the paired signals is calculated as Eq. 11.


(11) $$\Delta \,\varphi \,\left(t\right)=\arg\left(\frac{z_{1}\,\left(t\right)\,z_{2}^{*}\,\left(t\right)}{\left|z_{1}\,\left(t\right)\right|\,\left|z_{2}\,\left(t\right)\right|}\right)$$

Then, *PLI* can be defined as Eq. 12.


(12) P⁢L⁢I=|⟨sign⁢Δ⁢φ⁢(t)⟩|

where sign stands for signum function, |•| denotes the mean data and ⟨•⟩ indicate the absolute value. PLI ranges between 0 and 1. The larger the PLI value, the stronger the phase synchronization of the two EEG channels.

### Machine learning for classification

Three different machine learning models, support vector machine (SVM) (Chen et al., 2021), random forest (RF) (Murugappan et al., 2020), and K-nearest-neighbor (KNN) (Cover and Hart, 1967), were used to distinguish the differences between Old_GAD and Young_GAD in different rhythms.

(1) Support vector machine is a very common generalized linear classifier, which is mainly used for the classification of small sample data. Its ultimate goal is to find an optimal hyperplane to segment the samples. For linear inseparable problems, non-linear models need to be used to achieve accurate classification. The original data can be projected into a higher dimensional space by non-linear projection algorithm, so that the samples are linearly separable in the new space, and this process is achieved by defining an appropriate inner product kernel function. Common kernel functions include four kinds: linear kernel function, polynomial kernel function, Sigmoid kernel function and radial basis kernel function (RBF).
(2) Random forest is an ensemble algorithm based on a decision tree, which uses multiple trees to train and predict samples. The RF uses the Bagging method to generate an independent identically distributed training sample set for each decision tree, and the final classification result depends on the voting of all decision trees. Specifically, the idea of RF algorithm is that extracting *N* times from the original sample by bootstrap method, and m classification features are randomly selected from the total feature *M* each time (*m* ≤ *M*) to obtain N training sets. For each training set, a decision tree is used for training. For the test samples, the categories of new samples are determined by majority voting based on the classification results of *N* decision trees.
(3) K-nearest-neighbor algorithm is a supervised lazy machine learning algorithm. For the given test sample, the nearest k training samples in the training set are found based on some distance measurement, and then the prediction is made based on the information of these k “neighbors.” Usually, the category label that appears most in the k samples is selected as the prediction result.

In this study, a fivefold cross validation (fourfold samples for training and onefold sample for testing) with 10 repetitions was used to verify the generalization ability of the model, and the final evaluation result is the average of all test results. Moreover, the hyperparameters need to be set before the machine learning model is trained. In this study, SVM used the RBF kernel function to project samples into a high dimensional space, RF adopted 500 decision trees for ensemble classification, and KNN selected five nearest neighbors in training samples to test each sample and used euclidean distance measurement.

### Statistical analysis

In this study, One-way ANOVA was used to determine whether there were statistical differences in the EEG features between Young_GAD and Old_GAD. Specifically, if the final result p is less than 0.05, it could be regarded as having a significant statistical difference. All statistical analyses were implemented using MATLAB 2021b software (The MathWorks Inc., Natick, MA, USA).

## Results

**FIGURE 1:**
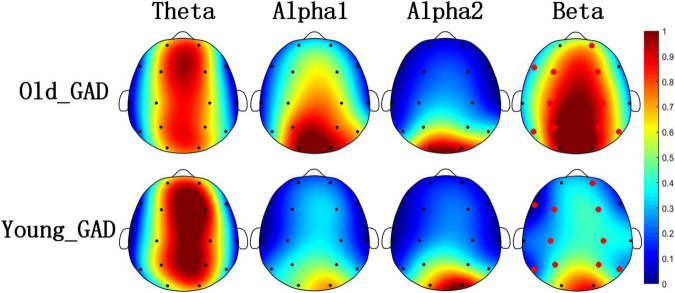
Brain topography of average absolute power (AP) of four rhythms (theta, alpha1, alpha2, and beta). The average AP of Old_GAD and Young_GAD have been normalized between 0 and 1, so they shared the same color bar. The red dots indicated the significantly differential electroencephalogram (EEG) channels between the two groups (*p* < 0.05).

**FIGURE 2:**
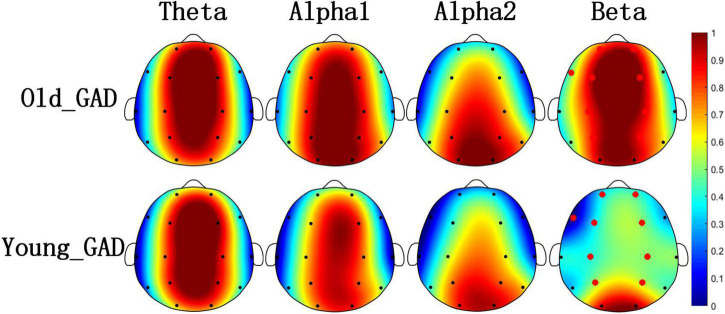
Brain topography of average fuzzy entropy (FE) of four rhythms (theta, alpha1, alpha2, and beta). The average FE of Old_GAD and Young_GAD have been normalized between 0 and 1, so they shared the same color bar. The red dots indicated the significantly differential electroencephalogram (EEG) channels between the Old_GAD and Young_GAD (*p* < 0.05).

Figures 1, 2 pointed out the results of the Old_GAD and Young_GAD regarding the AP and FE characteristics. In general, the results of AP and FE consistently depicted that the beta rhythm had significant differences compared with other rhythms (theta, alpha1, and alpha2). Specifically, the Old_GAD had higher AP and FE values on the beta rhythm than the Young_GAD, and it is most notably in the forehead, central and parietal regions, which were shown in the red electrode position.

**FIGURE 3:**
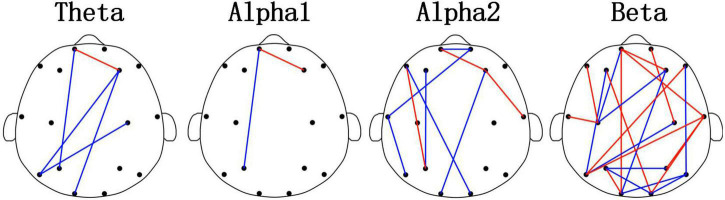
Differential functional connectivitys (FCs) (*p* < 0.05) between the Old_GAD and Young_GAD for four rhythms (theta, alpha1, alpha2, and beta). The red edge meant that the weight of the phase-lag-index (PLI) value in Old_GAD is higher than that in the Young_GAD, and the blue was the opposite.

Figure 3 and Table 3 showed the results of the PLI analysis. In theta and alpha2 rhythms, Young_GAD had more FCs with high values than the Old_GAD. But in the beta rhythm, Old_GAD had more FCs with high values than the Young_GAD. The number of FCs existing in the brain networks for lower rhythms (theta, alpha1, and alpha2) is much lower than that for higher rhythm (beta). The ratios were 13, 5, 24, and 58% for theta, alpha1, alpha2, and beta rhythms, which suggested that the beta band had the most difference between Young_GAD and Old_GAD. In addition, the distribution of these key FCs of beta rhythm was almost existed among all brain regions.

**TABLE 3:** Functional connectivity (FC) numbers of the Old_GAD and the Young_GAD in four rhythms.

	Theta	Alpha1	Alpha2	Beta
Old_GAD	1	1	3	12
Young_GAD	4	1	6	10

Table 4 showed the classification results of three common machine learning classifiers for the Old_GAD and Young_GAD. The classification accuracy for all features (including four rhythm features) were 99.67 ± 0.15, 98.26 ± 0.36, and 98.34 ± 0.32% with SVM, RF, and KNN models. We also calculated the accuracy of each rhythm. Every rhythm contained 152 EEG features, including 16 AP features, 16 FE features, and 120 PLI features. The highest accuracies for theta, alpha1, alpha2, and beta rhythms were 89.79 ± 1.03, 86.79 ± 0.71, 92.62 ± 0.35, and 99.46 ± 0.12%. In particular, the beta rhythm achieved the highest accuracy, which was much closer to the result of all features.

**TABLE 4:** Classification accuracies with different rhythms between Young_GAD and Old_GAD.

Features	SVM (%) (mean ± sd)	RF (%) (mean ± sd)	KNN (%) (mean ± sd)
All features	99.67 ± 0.15	98.26 ± 0.36	98.34 ± 0.32
Theta features	89.79 ± 1.03	82.35 ± 0.74	83.97 ± 0.64
Alpha1 features	86.79 ± 0.71	81.23 ± 1.02	82.35 ± 0.64
Alpha2 features	92.62 ± 0.35	86.32 ± 0.55	87.59 ± 0.81
Beta features	99.46 ± 0.12	95.82 ± 0.26	97.98 ± 0.17

## Discussion

This study investigated the EEG characteristics of the Young_GAD and Old_GAD based on resting-state EEG data from multiple dimensions (AP analysis, FE analysis, and PLI analysis) to reveal the neurodynamic mechanisms of the Young_GAD and Old_GAD. The main findings are as follows. Firstly, based on multiple perspective analyses of AP, FE, FC, and classification, the beta rhythm was significantly altered between the Young_GAD and Old_GAD. Secondly, the Old_GAD show obvious brain functional reorganization in beta rhythm. Thirdly, the extremely high classification accuracy of 99.46 ± 0.12% further confirmed the feasibility of classifying GAD into Young_GAD and Old_GAD. The results obtained will be analyzed in detail below.

### Significant aberration of the beta rhythm between Young_GAD and Old_GAD

Electroencephalogram rhythm contains rich information on brain neural activities, which has been widely used in the research and clinical applications of psychiatric diseases (Fenton et al., 1980; Grin-Yatsenko et al., 2009; Newson and Thiagarajan, 2019). In this study, Young_GAD and Old_GAD have significant differences in beta rhythm, and the important role of beta rhythm will be analyzed from multiple perspectives. (1) Compared with the Young_GAD patients, the Old_GAD patients had higher AP and FE values in beta rhythm in most brain regions, and there were significant statistical differences in the forehead, central, and parietal regions. The increased AP value of beta indicated that the brain activity is in a state of tension and neural nervousness, and the increased FE value indicated increased complexity (Al-Ezzi et al., 2022). Shen et al. (2022) has revealed that GAD patients have higher PSD and FE than healthy adults in beta rhythm. In this study, our results further pointed out that Old_GAD has higher anxiety levels and a more complex brain state in the beta rhythm. (2) The number of key FCs for beta rhythm accounted for 58% of the total number, which was much higher than theta, alpha1, and alpha2. While the changed number of FCs represented the alternation of regional coordination and cognitive function (Oathes et al., 2008) of brain regions (Greicius et al., 2009), it also reflected the reorganization of GAD in two age groups. (3) Beta rhythm achieved the highest classification accuracy (99.46%) compared with theta (89.79%), alpha1 (86.79%), and alpha2 (92,62%) rhythms, which meant that machine learning classification results could further support the specificity of beta rhythms in GAD patients over the two age groups.

The specificity of the beta rhythm in anxiety states (Grin-Yatsenko et al., 2009; Arsalan and Majid, 2022) and age-related changes (Gasser et al., 1988; Marciani et al., 1994; Cragg et al., 2011), respectively, it has received considerable attention in academic research and clinical applications. On the one hand, studies have been conducted to quantify the EEG characteristics of the beta rhythm for the assessment of anxiety. The Food and Drug Administration 4 (FDA4) approved the value of theta/beta as a biomarker for attention deficit and hyperactivity disorder (ADHD) (Newson and Thiagarajan, 2019). Al-Ezzi et al. (2022) showed that the FE value of social anxiety disorder (SAD) in beta rhythm was positively correlated with the Social Interaction Anxiety Scale (SIAS). On the other hand, more evidence also points to a high correlation between EEG characteristics of beta rhythm and age change. Al Zoubi et al. (2018) pointed out that the spectral flatness of beta is the most important predictor of age. Marciani et al. (1994) showed that the older the group, the greater the relative power value of the beta. In the present study, GAD patients of different ages showed significant differences in the EEG characteristics of beta rhythms. The Old_GAD had higher FE and AP values in several brain regions, with enhanced activity in this region reflecting increased cortical excitation (Porjesz et al., 2002) and metabolic activity (Yamada et al., 1995). In summary, the significant changes in the EEG characteristics of beta rhythm can help to better explain the neural mechanisms of Young_GAD and Old_GAD and provide basic theoretical support for subsequent studies of age-specific diagnosis and treatment of GAD.

Previous research has seldom focused on the EEG neural mechanism of different ages for GAD, and there is little precedent for GAD classification at different age levels with machine learning models. However, different age groups of GAD have been authenticated to play different symptoms (Javaid et al., 2022). Therefore, it is necessary to expand the corresponding EEG studies to provide a scientific foundation for the clinical triage of GAD patients of different ages. This study further revealed the neural mechanism of Young_GAD and Old_GAD.

### Functional reorganization overall the brain

Functional connectivity (FC) reflects the changes in connectivity between brain regions (Greicius et al., 2009) and is widely used to measure the effects of psychiatric disorders on brain structure and function (Fingelkurts et al., 2007; Tian et al., 2018). Pathology in the brain leads to a more random and disorganized network structure (Fingelkurts et al., 2007; Tian et al., 2018). Therefore, FC is an appropriate approach to gaining a comprehensive insight into the brain functional reorganization associated with psychiatric disorders. Psychiatric disorders interact with brain functional reorganization (Feinberg and Campbell, 2010). Psychiatric disorders are caused by brain reorganization, and brain reorganization advances psychiatric disorders. Alexander noted that depressed patients produce high-impact brain reorganization across multiple frequency ranges (Fingelkurts et al., 2006). Additionally, Scheinost et al. (2013) suggested that using fMRI neurofeedback to continuously reorganize brain networks in anxiety patients can non-invasively improve brain connectivity patterns and enhance the control of anxiety. Numerous studies have shown that different symptoms of psychiatric disorders also show diverse abnormal FC patterns (Meda et al., 2012). For example, GAD reduces FC in frontal and other brain regions (Shen et al., 2022). Patients with SAD have a limbic FC deficit, which is manifested by reduced frontal-occipital connectivity in resting-states (Xing et al., 2017). Patients with attention deficit show enhanced connectivity in frontal regions (Choi et al., 2013). In this study, PLI was used to estimate brain FC and key FC was further used to investigate mechanistic changes in GAD (Shen et al., 2022). We found that the key FC of Old_GAD patients showed significant reorganization throughout the brain compared to Young_GAD patients. Specifically, Old_GAD patients showed a significant decrease in key FC intensity in the low-frequency range (theta, alpha1, alpha2). It suggested that GAD patients showed a gradual decline in cognitive function with age, conforming to the general pattern of functional network changes in the aging brain (Oathes et al., 2008). Moreover, there was a significant enhancement and weakening of key FC in beta rhythm, which accounted for 58% of all connections in all rhythms. It suggested that Old_GAD patients had obvious whole-brain functional reorganization in beta rhythm.

### Evidence from the classification: Divide generalized anxiety disorder into Young_GAD and Old_GAD

Machine learning is widely used in medical research (Ancillon et al., 2022) and can assist in predicting and diagnosing psychiatric disorders by analyzing objective indicators of psychiatric disorder mechanisms (Park et al., 2021). In addition, studies have shown that EEG features can achieve the best performance as classification features compared to ECG, EDA, and RSP (Xu et al., 2015). Cai et al. (2018) extracted a variety of EEG features and combined them with feature selection algorithms, using KNN to obtain the highest classification accuracy (79.27%). Additionally, Shen et al. (2022) used an SVM model to classify healthy adults and GAD patients by EEG features and achieved an accuracy of 97%. In this study, EEG features of GAD patients of different ages were classified using machine learning (SVM, RF, and KNN models). The set of all rhythm features achieved an accuracy of 99.67%, demonstrating the effectiveness of applying machine learning to age classification diagnosis using EEG features. Moreover, the optimal feature set of the beta rhythm achieved the highest recognition performance of 99.46% among the four rhythms, which objectively reflected the specific performance of the beta rhythm among GAD patients of different ages.

Few studies used machine learning models to classify different ages of GAD, but satisfying results had been obtained from the age classification of healthy adults and GAD disease detection. Shen et al. (2022) showed that machine learning models can better distinguish between EEG features of GAD and healthy adults and have obtained the classification accuracy of 97% by SVM. Moezzi et al. (2019) have dichotomized the age of healthy adults by functional brain connectivity features and have obtained a classification accuracy of 93% by SVM. No studies have been conducted to classify EEG features of different ages of GAD by machine learning models yet. The present study obtained a high accuracy rate for the classification of Old_GAD and Young_GAD, which provides an objective scientific basis for the clinical triage of GAD patients by age.

### Limitations

This study inevitably has some limitations, which are listed as follows. Firstly, there were only 33 and 45 participants in the group of Young_GAD and Old_GAD, increasing the sample size will make the experiment more convincing. Secondly, the participants were limited to GAD patients and lacked comparison with the healthy control group, and it would be more meaningful to increase the sample of healthy people for the age classification study. Thirdly, this study used a 16-electrode EEG system with fewer electrode channels. If a high-density EEG system is used (for example, 64, 128 electrodes), richer experimental results may be obtained.

## Conclusion

In this study, we innovatively proposed to divide GAD patients into Young_GAD and Old_GAD according to age, and explored the similarities and differences of the neural mechanisms of Young_GAD and Old_GAD through EEG multidimensional features. It was found that Old_GAD and Young_GAD differed significantly in the beta rhythm EEG features, which could be used as neurological evidence for the division scheme. The feasibility of this division scheme was further validated by machine learning with a high accuracy of 99.67%. Overall, the present study can support the clinical field to diagnose and treat GAD patients by age division.

## Data availability statement

The raw data supporting the conclusions of this article will be made available by the authors, without undue reservation.

## Ethics statement

The studies involving human participants were reviewed and approved by Huzhou Third People’s Hospital. The patients/participants provided their written informed consent to participate in this study.

## Author contributions

GL conceived and designed the study. JW, JF, and GL performed the study, collected materials, and analyzed the results. JF and JW wrote the code and manuscript. JW and GL visualized the results. HL, YX, JL, and GL helped to coordinate the study and reviewed the manuscript. All authors contributed to the article and approved the submitted version.

## References

[B1] Aftanas L. I Pavlov S. V. (2005). Trait anxiety impact on posterior activation asymmetries at rest and during evoked negative emotions: EEG investigation. *Int. J. Psychophysiol.* 55 85–94. 10.1016/j.ijpsycho.2004.06.004 15598519

[B2] Al Zoubi O. Ki Wong C. Kuplicki R. T. Yeh H. W. Mayeli A. Refai H. (2018). Predicting age from brain EEG signals-A machine learning approach. *Front. Aging Neurosci.* 10:184. 10.3389/fnagi.2018.00184 30013472 PMC6036180

[B3] Al-Ezzi A. Al-Shargabi A. A. Shargie F. A. Zahary A. T. (2022). Complexity analysis of EEG in patients with social anxiety disorder using fuzzy entropy and machine learning techniques. *IEEE Access* 10 39926–39938. 10.1109/ACCESS.2022.3165199

[B4] Alotaibi N. Bakheet D. Konn D. Vollmer B. Maharatna K. (2022). Cognitive outcome prediction in infants with neonatal hypoxic-ischemic encephalopathy based on functional connectivity and complexity of the electroencephalography signal. *Front. Hum. Neurosci.* 15:795006. 10.3389/fnhum.2021.795006 35153702 PMC8830486

[B5] Altunoz U. Kokurcan A. Kirici S. Bastug G. Ozel-Kizil E. T. (2018). Clinical characteristics of generalized anxiety disorder: older vs. young adults. *Nordic J. Psychiatry* 72 97–102. 10.1080/08039488.2017.1390607 29065768

[B6] Ancillon L. Elgendi M. Menon C. (2022). Machine learning for anxiety detection using biosignals: a review. *Diagnostics* 12:1794. 10.3390/diagnostics12081794 35892505 PMC9332282

[B7] Arsalan A. Majid M. (2022). A study on multi-class anxiety detection using wearable EEG headband. *J. Ambient Intellig. Hum. Comput.* 13 5739–5749. 10.1007/s12652-021-03249-y

[B8] Bebbington P. Jacoby R. (1986). Psychiatric disorders in the elderly. *Can. J. Psychiatry Rev. Can. Psychiatr.* 56 387–397. 10.1177/070674371105600702 21835102

[B9] Blackmon K. Barr W. B. Carlson C. Devinsky O. DuBois J. Pogash D. (2011). Structural evidence for involvement of a left amygdala-orbitofrontal network in subclinical anxiety. *Psychiatry Res. Neuroimaging* 194 296–303. 10.1016/j.pscychresns.2011.05.007 21803551 PMC3544472

[B10] Buff C. Schmidt C. Brinkmann L. Gathmann B. Tupak S. Straube T. (2018). Directed threat imagery in generalized anxiety disorder. *Psychol. Med.* 48 617–628. 10.1017/S0033291717001957 28735579

[B11] Cai H. Han J. Chen Y. Sha X. Wang Z. Hu B. (2018). A pervasive approach to eeg-based depression detection. *Complexity* 2018:5238028.

[B12] Chen C. Yu X. Belkacem A. N. Lu L. Li P. Zhang Z. (2021). EEG-based anxious states classification using affective BCI-based closed neurofeedback system. *J. Med. Biol. Eng.* 41 155–164. 10.1007/s40846-020-00596-7 33564280 PMC7862980

[B13] Chen W. Wang Z. Xie H. Yu W. (2007). Characterization of surface EMG signal based on fuzzy entropy. *IEEE Trans. Neural Syst. Rehabil. Eng.* 15 266–272. 17601197 10.1109/TNSRE.2007.897025

[B14] Choi J. Jeong B. Lee S. W. Go H. J. (2013). Aberrant development of functional connectivity among resting state-related functional networks in medication-naive ADHD children. *PLoS One* 8:e83516. 10.1371/journal.pone.0083516 24386219 PMC3873390

[B15] Choi J. Lim E. Park M. G. Cha W. (2020). Assessing the retest reliability of prefrontal EEG markers of brain rhythm slowing in the eyes-closed resting state. *Clin. EEG Neurosci.* 51 348–356. 10.1177/1550059420914832 32253926

[B16] Clancy K. J. Andrzejewski J. A. Simon J. Ding M. Schmidt N. B. Li W. (2020). Posttraumatic stress disorder is associated with alpha dysrhythmia across the visual cortex and the default mode network. *ENEURO* 7:ENEURO.0053-20.2020. 10.1523/ENEURO.0053-20.2020 32690671 PMC7405069

[B17] Cover T. Hart P. (1967). Nearest neighbor pattern classification. *IEEE Trans. Inform. Theory* 13 21–27. 10.1109/TIT.1967.1053964

[B18] Cragg L. Kovacevic N. McIntosh A. R. Poulsen C. Martinu K. Leonard G. (2011). Maturation of EEG power spectra in early adolescence: a longitudinal study. *Dev. Sci.* 14 935–943. 10.1111/j.1467-7687.2010.01031.x 21884309

[B19] Feinberg I. Campbell I. G. (2010). Sleep EEG changes during adolescence: an index of a fundamental brain reorganization. *Brain Cogn.* 72 56–65. 10.1016/j.bandc.2009.09.008 19883968

[B20] Feng X. Forbes E. E. Kovacs M. George C. J. Lopez-Duran N. L. Fox N. A. (2012). Children’s depressive symptoms in relation to EEG frontal asymmetry and maternal depression. *J. Abnorm. Child Psychol.* 40 265–276. 10.1007/s10802-011-9564-9 21894523 PMC3262060

[B21] Fenton G. W. Fenwick P. B. Dollimore J. Dunn T. L. Hirsch S. R. (1980). EEG spectral analysis in schizophrenia. *Br. J. Psychiatry* 136 445–455. 7388249 10.1192/bjp.136.5.445

[B22] Fingelkurts A. A. Fingelkurts A. A. Rytsälä H. Suominen K. Isometsä E. Kähkönen S. (2006). Composition of brain oscillations in ongoing EEG during major depression disorder. *Neurosci. Res.* 56 133–144. 10.1016/j.neures.2006.06.006 16860895

[B23] Fingelkurts A. A. Fingelkurts A. A. Rytsälä H. Suominen K. Isometsä E. Kähkönen S. (2007). Impaired functional connectivity at EEG alpha and theta frequency bands in major depression. *Hum. Brain Mapp.* 28 247–261. 16779797 10.1002/hbm.20275 PMC6871285

[B24] Flint A. J. (2005). Generalised anxiety disorder in elderly patients : epidemiology, diagnosis and treatment options. *Drugs Aging* 22 101–114. 10.2165/00002512-200522020-00002 15733018

[B25] Flint A. J. (2009). Late-life generalized anxiety: the constraint of categorization. *Am. J. Geriatr. Psychiatry* 17 441–444. 10.1097/JGP.0b013e3181a2fbd4 19461255

[B26] Fusina F. Marino M. Spironelli C. Angrilli A. (2022). Ventral attention network correlates with high traits of emotion dysregulation in community women - a resting-state EEG study. *Front. Hum. Neurosci.* 16:895034. 10.3389/fnhum.2022.895034 35721362 PMC9205637

[B27] Gasser T. Verleger R. Bächer P. Sroka L. (1988). Development of the EEG of school-age children and adolescents. I. Analysis of band power. *Electroencephalogr. Clin. Neurophysiol.* 69 91–99. 2446839 10.1016/0013-4694(88)90204-0

[B28] Gayete S. Giné A. Miret M. Ayuso-Mateos J. L. Haro J. M. Olaya B. (2020). Cognitive function associated with different diagnoses of anxiety disorders over the lifespan: results from a Spanish representative sample. *J. Anxiety Disord.* 75:102296. 10.1016/j.janxdis.2020.102296 32866758

[B29] Greicius M. D. Supekar K. Menon V. Dougherty R. F. (2009). Resting-state functional connectivity reflects structural connectivity in the default mode network. *Cereb. Cortex* 19 72–78. 18403396 10.1093/cercor/bhn059 PMC2605172

[B30] Grillon C. Buchsbaum M. S. (1987). EEG topography of response to visual stimuli in generalized anxiety disorder. *Electroencephalogr. Clin. Neurophysiol.* 66 337–348. 10.1016/0013-4694(87)90031-9 2435513

[B31] Grin-Yatsenko V. A. Baas I. Ponomarev V. A. Kropotov J. D. (2009). EEG power spectra at early stages of depressive disorders. *J. Clin. Neurophysiol.* 26 401–406. 19952564 10.1097/WNP.0b013e3181c298fe

[B32] Hilbert K. Lueken U. Beesdo-Baum K. (2014). Neural structures, functioning and connectivity in Generalized Anxiety Disorder and interaction with neuroendocrine systems: a systematic review. *J. Affect. Disord.* 158 114–126. 10.1016/j.jad.2014.01.022 24655775

[B33] Huang Z. Li Y. Bianchi M. T. Zhan S. Jiang F. Li N. (2018). Repetitive transcranial magnetic stimulation of the right parietal cortex for comorbid generalized anxiety disorder and insomnia: a randomized, double-blind, sham-controlled pilot study. *Brain Stimul.* 11 1103–1109. 10.1016/j.brs.2018.05.016 29871798

[B34] Huang Z. Zhan S. Chen C. Li N. Ding Y. Hou Y. (2019). The effect of insomnia on cortical excitability in patients with generalized anxiety disorder. *Front. Psychiatry* 9:755. 10.3389/fpsyt.2018.00755 30687140 PMC6335338

[B35] Imperatori C. Farina B. Adenzato M. Valenti E. M. Murgia C. Marca G. D. (2019). Default mode network alterations in individuals with high-trait-anxiety: an EEG functional connectivity study. *J. Affect. Disord.* 246 611–618. 10.1016/j.jad.2018.12.071 30605880

[B36] Javaid H. Kumarnsit E. Chatpun S. (2022). Age-Related Alterations in EEG Network Connectivity in Healthy Aging. *Brain Sci.* 12:218. 10.3390/brainsci12020218 PMC8870284 35203981

[B37] Jesulola E. Sharpley C. F. Bitsika V. Agnew L. L. Wilson P. (2015). Frontal alpha asymmetry as a pathway to behavioural withdrawal in depression: research findings and issues. *Behav. Brain Res.* 292 56–67. 10.1016/j.bbr.2015.05.058 26051816

[B38] Knyazev G. G. Savostyanov A. N. Levin E. A. (2004). Alpha oscillations as a correlate of trait anxiety. *Int. J. Psychophysiol.* 53 147–160. 15210292 10.1016/j.ijpsycho.2004.03.001

[B39] Le Roux H. Gatz M. Wetherell J. L. (2005). Age at onset of generalized anxiety disorder in older adults. *Am. J. Geriatr. Psychiatry* 13 23–30. 10.1097/00019442-200501000-00005 15653937

[B40] Lenze E. J. Rollman B. L. Shear M. K. Dew M. A. Pollock B. G. Ciliberti C. (2009). Escitalopram for older adults with generalized anxiety disorder a randomized controlled trial. *J. Am. Med. Assoc.* 301 295–303. 10.1001/jama.2008.977 PMC2840403 19155456

[B41] Li W. Zhang W. Jiang Z. Zhou T. Xu S. Zou L. (2022). Source localization and functional network analysis in emotion cognitive reappraisal with EEG-fMRI integration. *Front. Hum. Neurosci.* 16:960784. 10.3389/fnhum.2022.960784 36034109 PMC9411793

[B42] Mah L. Szabuniewicz C. Fiocco A. J. (2016). Can anxiety damage the brain? *Curr. Opin. Psychiatry* 29 56–63. 26651008 10.1097/YCO.0000000000000223

[B43] Marciani M. G. Maschio M. Spanedda F. Caltagirone C. Gigli G. L. Bernardi G. (1994). Quantitative EEG evaluation in normal elderly subjects during mental processes: age-related changes. *Int. J. Neurosci.* 76 131–140. 10.3109/00207459408985998 7960462

[B44] Massullo C. Carbone G. A. Farina B. Panno A. Capriotti C. Giacchini M. (2020). Dysregulated brain salience within a triple network model in high trait anxiety individuals: a pilot EEG functional connectivity study. *Int. J. Psychophysiol.* 157 61–69. 10.1016/j.ijpsycho.2020.09.002 32976888

[B45] Meda S. A. Gill A. Stevens M. C. Lorenzoni R. P. Glahn D. C. Calhoun V. D. (2012). Differences in resting-state functional magnetic resonance imaging functional network connectivity between schizophrenia and psychotic bipolar probands and their unaffected first-degree relatives. *Biol. Psychiatry* 71 881–889. 22401986 10.1016/j.biopsych.2012.01.025 PMC3968680

[B46] Miloyan B. Byrne G. J. Pachana N. A. (2014). Age-related changes in generalized anxiety disorder symptoms. *Int. Psychogeriatr.* 26 565–572. 24405581 10.1017/S1041610213002470

[B47] Moezzi B. Pratti L. M. Hordacre B. Graetz L. Berryman C. Lavrencic L. M. (2019). Characterization of young and old adult brains: an EEG functional connectivity analysis. *Neuroscience* 422 230–239. 10.1016/j.neuroscience.2019.08.038 31806080

[B48] Mohlman J. Eldreth D. A. Price R. B. Staples A. M. Hanson C. (2017). Prefrontal-limbic connectivity during worry in older adults with generalized anxiety disorder. *Aging Ment. Health* 21 426–438. 10.1080/13607863.2015.1109058 26566020

[B49] Mporas I. Tsirka V. Zacharaki E. I. Koutroumanidis M. Richardson M. Megalooikonomou V. (2015). Seizure detection using EEG and ECG signals for computer-based monitoring, analysis and management of epileptic patients. *Expert Syst. Appl.* 42 3227–3233. 10.1016/j.eswa.2014.12.009

[B50] Murugappan M. Alshuaib W. Bourisly A. K. Khare S. K. Sruthi S. Bajaj V. (2020). Tunable Q wavelet transform based emotion classification in Parkinson’s disease using electroencephalography. *PLoS One* 15:e0242014. 10.1371/journal.pone.0242014 33211717 PMC7676721

[B51] Newson J. J. Thiagarajan T. C. (2019). EEG frequency bands in psychiatric disorders: a review of resting state studies. *Front. Hum. Neurosci.* 12:521. 10.3389/fnhum.2018.00521 30687041 PMC6333694

[B52] Nikias C. L. Petropulu A. P. (1993). *Higher Order Spectra Analysis: A Non-Linear Signal Processing Framework.* Hoboken, NJ: Prentice Hall.

[B53] Oathes D. J. Ray W. J. Yamasaki A. S. Borkovec T. D. Castonguay L. G. Newman M. G. (2008). Worry, generalized anxiety disorder, and emotion: evidence from the EEG gamma band. *Biol. Psychol.* 79 165–170. 10.1016/j.biopsycho.2008.04.005 18499328 PMC2597009

[B54] Olatunji B. O. Wolitzky-Taylor K. B. Sawchuk C. N. Ciesielski B. G. (2010). Worry and the anxiety disorders: a meta-analytic synthesis of specificity to GAD. *Appl. Prevent. Psychol.* 14 1–24.

[B55] Pang J. Tang X. Li H. Hu Q. Cui H. Zhang L. (2019). Altered interoceptive processing in generalized anxiety disorder a heartbeat - evoked potential research. *Front. Psychiatry* 10:616. 10.3389/fpsyt.2019.00616 31543837 PMC6739601

[B56] Papadimitriou G. N. Kerkhofs M. Kempenaers C. Mendlewicz J. (1988). EEG sleep studies in patients with generalized anxiety disorder. *Psychiatry Res.* 26 183–190. 3237912 10.1016/0165-1781(88)90073-x

[B57] Park S. M. Jeong B. Oh D. Y. Choi C.-H. Jung H. Y. Lee J.-Y. (2021). Identification of major psychiatric disorders from resting-state electroencephalography using a machine learning approach. *Front. Psychiatry* 12:707581. 10.3389/fpsyt.2021.707581 34483999 PMC8416434

[B58] Piho L. Tjahjadi T. (2020). A mutual information based adaptive windowing of informative EEG for emotion recognition. *IEEE Trans. Affect. Comput.* 11 722–735.

[B59] Porjesz B. Almasy L. Edenberg H. J. Wang K. Chorlian D. B. Foroud T. (2002). Linkage disequilibrium between the beta frequency of the human EEG and a GABAA receptor gene locus. *Proc. Natl. Acad. Sci. U.S.A.* 99 3729–3733. 10.1073/pnas.052716399 11891318 PMC122592

[B60] Pradhan N. Dutt D. N. (1994). Data compression by linear prediction for storage and transmission of EEG signals. *Int. J. Bio Med. Comput.* 35 207–217. 10.1016/0020-7101(94)90076-0 8005713

[B61] Pull C. B. (1995). [DSM-IV]. *L’Encephale* 21 15–20. 8582301

[B62] Santomauro D. F. Mantilla Herrera A. M. Shadid J. Zheng P. Ashbaugh C. Pigott D. M. (2021). Global prevalence and burden of depressive and anxiety disorders in 204 countries and territories in 2020 due to the COVID-19 pandemic. *Lancet* 398 1700–1712. 10.1016/S0140-6736(21)02143-7 34634250 PMC8500697

[B63] Scheinost D. Stoica T. Saksa J. Papademetris X. Constable R. T. Pittenger C. (2013). Orbitofrontal cortex neurofeedback produces lasting changes in contamination anxiety and resting-state connectivity. *Transl. Psychiatry* 3:e250. 10.1038/tp.2013.24 23632454 PMC3641411

[B64] Seoane S. Ezama L. Janssen N. (2022). Daily-life physical activity of healthy young adults associates with function and structure of the hippocampus. *Front. Hum. Neurosci.* 16:790359. 10.3389/fnhum.2022.790359 PMC8963905 35360290

[B65] Shen Z. X. Li G. Fang J. Zhong H. Wang J. Sun Y. (2022). Aberrated multidimensional EEG characteristics in patients with generalized anxiety disorder: a machine-learning based analysis framework. *Sensors* 22:5420. 10.3390/s22145420 35891100 PMC9320264

[B66] Skoog I. (2011). Psychiatric disorders in the elderly. *Can. J. Psychiatry Rev. Can. Psychiatr.* 56 387–397. 10.1177/070674371105600702 21835102

[B67] Smith E. E. Zambrano-Vazquez L. Allen J. J. B. (2016). Patterns of alpha asymmetry in those with elevated worry, trait anxiety, and obsessive-compulsive symptoms: a test of the worry and avoidance models of alpha asymmetry. *Neuropsychologia* 85 118–126. 10.1016/j.neuropsychologia.2016.03.010 26970143

[B68] Stam C. J. Nolte G. Daffertshofer A. (2007). Phase lag index: assessment of functional connectivity from multi channel EEG and MEG with diminished bias from common sources. *Hum. Brain Mapp.* 28 1178–1193. 10.1002/hbm.20346 17266107 PMC6871367

[B69] Steiger A. Pawlowski M. (2019). Depression and sleep. *Int. J. Mol. Sci.* 20:607. 10.3390/ijms20030607 30708948 PMC6386825

[B70] Sylvers P. Lilienfeld S. O. LaPrairie J. L. (2011). Differences between trait fear and trait anxiety: implications for psychopathology. *Clin. Psychol. Rev.* 31 122–137. 20817337 10.1016/j.cpr.2010.08.004

[B71] Thorp S. R. Ayers C. R. Nuevo R. Stoddard J. A. Sorrell J. T. Wetherell J. L. (2009). Meta-analysis comparing different behavioral treatments for late-life anxiety. *Am. J. Geriatr. Psychiatry* 17 105–115. 10.1097/JGP.0b013e31818b3f7e 19155744 PMC2794407

[B72] Tian L. Li Q. Wang C. Yu J. (2018). Changes in dynamic functional connections with aging. *Neuroimage* 172 31–39. 10.1016/j.neuroimage.2018.01.040 29414496

[B73] Tyrer P. Baldwin D. (2006). Generalised anxiety disorder. *Lancet* 368 2156–2166. 10.1016/S0140-6736(06)69865-6 17174708

[B74] Vogelzangs N. Beekman A. T. F. de Jonge P. Penninx B. W. J. H. (2013). Anxiety disorders and inflammation in a large adult cohort. *Transl. Psychiatry* 3:e249. 10.1038/tp.2013.27 23612048 PMC3641413

[B75] Wang Y. Chai F. Zhang H. Liu X. Xie P. Zheng L. (2016). Cortical functional activity in patients with generalized anxiety disorder. *BMC Psychiatry* 16:217. 10.1186/s12888-016-0917-3 27388467 PMC4936202

[B76] Wetherell J. L. Gatz M. Craske M. G. (2003). Treatment of generalized anxiety disorder in older adults. *J. Consul. Clin. Psychol.* 71 31–40. 10.1037/0022-006X.71.1.31 12602423

[B77] Xing M. Tadayonnejad R. MacNamara A. Ajilore O. DiGangi J. Phan K. L. (2017). Resting-state theta band connectivity and graph analysis in generalized social anxiety disorder. *Neuroimage Clin.* 13 24–32. 10.1016/j.nicl.2016.11.009 27920976 PMC5126152

[B78] Xu L. Xu H. Ding H. Li J. Wang C. (2021). Intrinsic network brain dysfunction correlates with temporal complexity in generalized anxiety disorder and panic disorder. *Front. Hum. Neurosci.* 15:647518. 10.3389/fnhum.2021.647518 34335204 PMC8319536

[B79] Xu Q. Nwe T. L. Guan C. (2015). Cluster-based analysis for personalized stress evaluation using physiological signals. *IEEE J. Biomed. Health Inform.* 19 275–281. 10.1109/JBHI.2014.2311044 25561450

[B80] Yamada M. Kimura M. Mori T. Endo S. (1995). [EEG power and coherence in presenile and senile depression. Characteristic findings related to differences between anxiety type and retardation type]. *Nihon Ika Daigaku Zasshi* 62 176–185. 10.1272/jnms1923.62.176 7775654

